# Is Undernutrition Associated With Deterioration of Outcomes in the Pediatric Intensive Care Unit (PICU): Systematic and Meta-Analysis Review

**DOI:** 10.3389/fped.2022.769401

**Published:** 2022-05-04

**Authors:** Maram S. Albadi, Khlood Bookari

**Affiliations:** ^1^Clinical Nutrition Department, Maternity and Children Hospital, Medina, Saudi Arabia; ^2^Clinical Nutrition Department, Faculty of Applied Medical Sciences, Taibah University, Medina, Saudi Arabia

**Keywords:** nutrition status, mortality incidence, length of stay, the need for and length of time on mechanical ventilation, pediatric intensive care unit, undernutrition

## Abstract

**Background and aim:**

Undernutrition (UN) may negatively impact clinical outcomes for hospitalized patients. The relationship between UN status at pediatric intensive care unit (PICU) admission and clinical outcomes is still not well-reported. This systematic meta-analysis review evaluated the impact of UN at admission to PICU on clinical outcomes, including mortality incidence, length of stay (LOS), and the need for and length of time on mechanical ventilation (MV).

**Methods:**

A search was conducted using relevant and multi-medical databases from inception until January 2022. We considered studies that examined the link between UN at PICU admission and clinical outcomes in patients aged 18 years or younger. Pooled risk difference estimates for the PICU outcomes were calculated using a random-effects model.

**Result:**

There were a total of 10,638 patients included in 17 observational studies; 8,044 (75.61%) and 2,594 (24.38%) patients, respectively, were normal-nourished (NN) and undernourished (UN). In comparison to NN patients, UN patients had a slightly higher risk of mortality (RD = 0.02, *P* = 0.05), MV usage (RD = 0.05, *P* = 0.02), and PICU LOS (RD = 0.07, *P* = 0.007). While the duration of MV was significantly longer in UN than in NN (RD = 0.13, *P* < 0.0001). Sensitivity analysis of UN classification cohorts with a z-score < -2 or in the 5%, patetints age up to 18 years, and mixed diagnose for PICU admission demonstrated a 6-fold increase in the probability of PICU LOS in UN patients compared to NN patients (RD = 0.06, 95% CI = 0.01, 0.12). UN patients have a higher risk of MV usage RD = 0.07, 95% CI = 0.00, 0.14) in studies involving cohorts with a mixed primary diagnosis for PICU admission.

**Conclusion:**

In PICU, UN is linked to mortality incidence, longer PICU stay, MV usage, and duration on MV. The primary diagnosis for PICU admission may also influence clinical outcomes. Determining the prevalence of UN in hospitalized patients, as well as the subgroups of patients diagnosed at the time of admission, requires more research. This may help explain the relationship between nutritional status and clinical outcomes in PICU patients.

## Introduction

Malnutrition and undernutrition (UN) are commonly used interchangeably, although they are not synonyms. According to the World Health Organization (WHO), malnutrition is described as an imbalance in someone's energy and nutrient intake, which can occur at either end of the weight spectrum (1). In contrast, UN is a term that refers to nutritional inadequacies in an individual's energy and nutrient intake and absorption (2). There are four main types of UN: wasting, stunting, underweight, and micronutrient deficiencies (3). Wasting is characterized by low weight for height. It frequently implies rapid weight reduction, although it can also be sustained. It usually occurs when an individual has not consumed enough or has been sick frequently (3). If left untreated, wasting in children increases the chance of mortality. Stunting is recognized as a short height for age. It is the outcome of chronic or repeated malnutrition, which is frequently connected with poverty, poor prenatal health and nutrition, frequent illness, and/or improper early life feeding and care. Children who are stunted cannot realize their full physical and cognitive capabilities. Underweight is characterized as having an abnormally low weight for one's age. Underweight children may be stunted, wasted, or both. Micronutrient deficiencies are a deficiency of vitamins and minerals that are necessary for the body to operate properly, including the production of enzymes, hormones, and other compounds essential for growth and development (3).

Undernutrition is more prevalent in women, infants, children, and adolescents (3). Children who are severely ill are also in danger of nutritional inadequacy due to the disease itself and the failure to deliver nutrients (4, 5). Although UN is widespread among hospitalized children, especially those admitted to pediatric intensive care units (PICUs), it is underreported (6). The reported prevalence of UN in hospitalized children ranges from 2.5 to 51% (7). It varies depending on the population investigated, clinical settings, characteristics, and categorization systems (7). In addition, this discrepancy stems from the fact that there is no universally accepted definition of pediatric UN (7). There has been a link established in a number of studies between UN and increased mortality and hospitalization duration and an increased number of organ dysfunctions and complications (8, 9). Pediatric patients who are malnourished and admitted to PICUs are at an increased risk of infection and death (10). Moreover, the UN is a growing concern due to its association with poor PICU outcomes (4, 5, 7, 11). Underweight is linked explicitly with an increased incidence of death (4, 7, 8), length of stay (LOS) (9), and duration of mechanical ventilation (MV) (5). Given that UN upon admission or worsening of the nutritional status during hospitalization has a detrimental effect on clinical outcomes and increases healthcare expenses, better identification and documentation of the impact of UN on critically ill children is warranted.

To date, the effect of malnutrition on critically ill children has been studied by only a few previous systematic studies (12). The study conducted by Costa et al. (13) investigated the relationship between UN and PICU outcomes. It revealed that undernourished patients had a longer MV duration. However, the study found inconclusive evidence of a connection between prolonged PICU LOS and mortality (13). Alipoor et al. undertook a systematic evaluation of observational data and a meta-analysis in 2019 for their research into obesity in critically ill children. Compared to normal-weight pediatric patients, they found that obese pediatric patients had a higher risk of mortality and a longer stay in the hospital. They also report a non-statistically significant connection between prolonged PICU LOS and decreased MV length in the obese pediatric patients' group, which they attribute to a lack of statistical significance (14). A more recent review defined the total impact of malnutrition in critically unwell children using PICU admission body mass index (BMI). They evaluated mortality, LOS, and MV length in underweight and overweight PICU patients. They also assessed the impact of BMI on PICU outcomes across nations with varying socioeconomic levels (as defined by the World Bank). However, previous studies characterized both undernutrition and overnutrition by combining anthropometric parameters such as weight for age, BMI, and skin folds with laboratory data (e.g., albumin and C-reactive protein) or by focusing primarily on PICU entrance BMI (12). To the best of our knowledge, there are limited studies that focus on the association between UN in PICU only and outcomes in critically ill children. To address this gap in the literature, we undertook this study to update and improve the evidence foundation regarding the association between UN and children's PICU outcomes, including mortality incidence, LOS in the PICU, the necessity for MV, and duration of MV use. A thorough examination of the currently available data was also revealed.

## Methodology

Moher et al. (15) recommended using the Preferred Reporting Items for Systematic Reviews and Meta-analyses (PRISMA) flowchart to identify evidence-based research protocols in the context of a systematic review and meta-analysis.

### Literature Search

The National Library of Medicine, SpringerLink PubMed, Excerpta Medica Database (Embase), Cumulative Index of Nursing and Allied Health Literature (CINAHL), Cochrane Library, Web of Science, and ScienceDirect were searched using the search terms such as “nutrition status” or “undernutrition” or “malnutrition” or “nutrition assessment” and “pediatric intensive care unit” or “PICU” or “children intensive care.” The publication dates of the observational studies were restricted to 2012–January 2022. The results were filtered to include only those studies published in the English language that were peer-reviewed and for which the full text of the articles was available. Furthermore, references were reviewed to identify any additional studies.

### Inclusion Criteria and Study Selection

The primary criterion in searching the selected databases was that the articles should be observational studies, whether prospective or retrospective, involving children of both genders who were younger than 19 years of age. The studies had to assess one or more of the following outcomes: mortality rate, the LOS in the PICU, and the need and length of MV usage. Subsequently, the abstracts were screened against the inclusion and exclusion criteria. Finally, the full-text versions of papers that met the inclusion criteria were reviewed. Table 1 shows the inclusion criteria in greater detail.

**Table 1:** Inclusion and exclusion criteria.

**Variable**	**Inclusion criteria**	**Exclusion criteria**
Population	- Humans - Children aged 0–18 years - Full-term infants - Both genders	- Animals and laboratory-based studies - Adults ≥ 19 years old - Preterm infants
Exposure	UN status defined by WHO: - BMI or W/H - MUAC - Skin fold thickness	- Overnutrition status - Unavailable anthropometric data (i.e., weight and height) - Nutrition status classified using a different tool (e.g., Screening Tool for the Assessment of Malnutrition in Paediatrics)
Comparison	NN status	- Different grades of UN - Overnutrition status
Outcomes	- Mortality rate - LOS in PICU - Need for MV - Prolonged use of MV	- Infection rate - LOS in hospital - Morbidity rate - Nutritional intervention difference
Timing	Anthropometric measurement performed within 24 h of admission	Anthropometric measurement performed after 24 h
Setting	PICU	Admission to any ward other than the PICU

*PICU, pediatric intensive care unit; MV, mechanical ventilation; LOS, length of stay; UN, undernutrition; WHO, World Health Organization; BMI, body mass index; W/H, weight for height; MUAC, middle-upper arm circumference*.

### Data Extraction

Data and outcomes relevant to the research were extracted from the included studies. The study design, country, number of participants (total and within each group), population characteristics (i.e., mean age, gender ratio, and diagnosis), the definition of UN level, and any predefined model results were collected and sorted into tables.

### Study Quality Assessment

The risk of bias was assessed for all the studies included. For this purpose, the modified National Heart, Lung, and Blood Institute (NHLBI) tool was used to assess the quality of the eligible studies. This quality assessment tool has been used in previously published systematic reviews (16). The tool evaluates the risk of bias and internal validity of three aspects: selection bias, information bias, and measurement or confounding bias. Table 2 presents the questions asked in greater detail (NHLBI, n/d). Each study was evaluated against the items listed in the tool, and the answer was recorded as yes, no, not applicable, or unclear. This provided an overall quality rating for each study, and the studies were then classified into three levels according to the yes answer count: poor (score: 0–4), fair (score: 5–8), and good (score: 9–12).

**Table 2:** The modified National Heart, Lung, and Blood Institute (NHLBI) quality assessment tool.

**Question**	**Answer**
1. Was the research question or objective in this paper clearly stated? 2. Was the study population clearly specified and defined? 3. Was the participation rate of eligible persons at least 50%? 4. Were all the subjects selected or recruited from the same or similar populations (including the same time period)? 5. Was a sample size justification, power description, or variance and effect estimates provided? 6. For the analyses in this paper, were the exposure(s) of interest measured prior to the outcome(s) being measured? 7. Was the timeframe sufficient so that one could reasonably expect to see an association between exposure and outcome if it existed? 8. For exposures that can vary in amount or level, did the study examine different levels of the exposure as related to the outcome (e.g., categories of exposure, or exposure measured as a continuous variable)? 9. Were the exposure measures (independent variables) clearly defined, valid, reliable and implemented consistently across all study participants? 10. Were the outcome measures (dependent variables) clearly defined, valid, reliable and implemented consistently across all study participants? 11. Were the outcome assessors blinded to the exposure status of participants? 12. Were key potential confounding variables measured and adjusted statistically for their impact on the relationship between exposure(s) and outcome(s)?	

*Possible answers: N, no; NA, not applicable; U, unclear; Y, yes*.

### Data Analysis

Nutritional status was *a priori* evaluated by using the following anthropometrics: W/H for infants aged <2 years, BMI for children aged between 2 and 18 years, middle-upper arm circumference (MUAC), or skinfold thickness (SFT). WHO or CDC liner growth charts were applied for classification: UN (z < −2) and normal nutrition (z >-2 to 1) (17). However, due to variations reported in UN groups in some studies (e.g., mild, moderate, and severe grade UN), a pooled percentage from both groups was used (18). In some studies, it was necessary to extract the data from a figure. The methods of extracting data from the figures outlined by Rich et al. (19) were followed. All statistical analysis was conducted using Review Manager software version 5 (Cochrane Collaboration, Copenhagen, Denmark).

Each of the outcomes was pooled using the inverse variance random-effects model to account for heterogeneity between studies. We shifted to the fixed-effects model analysis setting when the random effects model test was estimating a heterogeneous. All results were presented in forest plots. The categories of mortality, need for MV incidence in UN, and healthy nutrition were dichotomies pooled separately and were expressed as pooled risk difference (RD) with a 95% confidence interval (95% CI). Continuous outcomes, including PICU LOS and MV duration, were also pooled, and pooled values were expressed as an adjusted standardized mean difference (SMD). The longer stay in PICU and MV event numbers from selected studies for UN vs. normal nutrition were also pooled and were reposted as pooled RD 95% CI.

All the study sources were included in the clinical heterogeneity examinations by calculating the *I*^2^ and Cochran's Q-test statistical tests. Heterogeneity was considered unimportant when *I*^2^ ≤ 30%, moderate (30–50%), substantial (50–75%), and >75% is considerable, and homogeneity is significant if the *P*-value of Cochran's Q-test was ≧0.05 (20). Two heterogeneity analysis methods are used in this study to ensure that all studies are estimating the same average effect size.

First, publications with a non-significant outcome were eliminated to drop heterogeneity to lower than 50%. Then, if at least two studies are available in each subgroup, we conducted the whole included studies using the subgroup heterogeneity analysis approach. The inquiry was impacted at the economic nation level, and the subgroup approach was utilized to compare pooled data from studies in developed and developing countries. Patients with mixed and specific diagnoses were pooled in research to evaluate the impact on outcomes. Furthermore, another subgroup was conducted, such as the method of defining UN states in included studies (≤ -2 z_score_ or ≤ 5th percentile vs. ≤ -1 z_score_), the higher allowed age range (≤ 18 years vs. ≤ 16years), the cutoff for a lengthy stay in LOS (≧7 days vs. ≧3 days), and MV (≧7 days vs. ≧5 days).

The funnel plots are visually inspected and used to evaluate the publication bias if at least ten studies are included (21). RevMan software was used to run Egger's regression. Publication bias is considered in asymmetry funnel plots (20).

## Results

### Search Results

**Figure 1:**
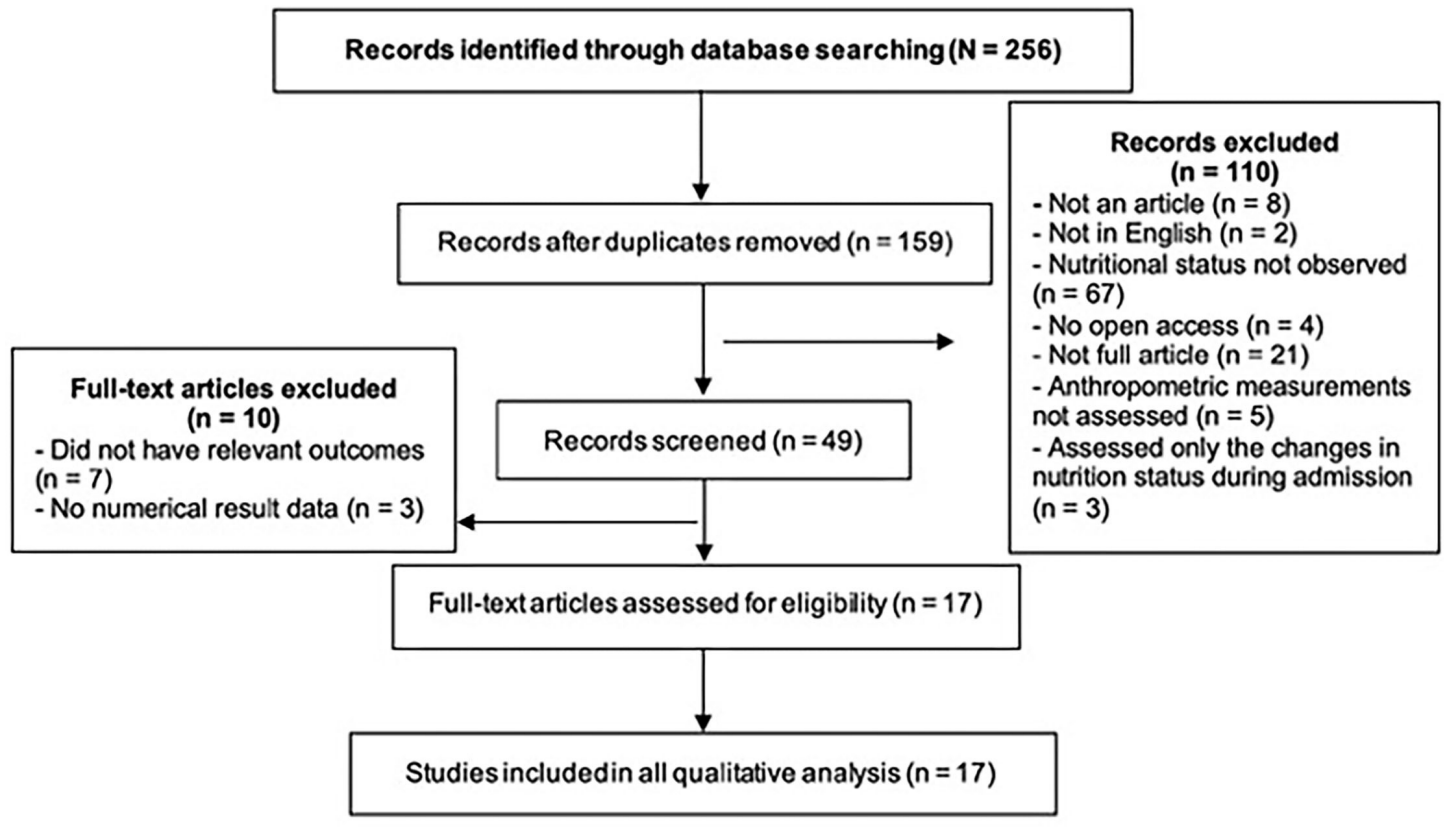
PRISMA flow diagram employed in the literature search process to identify articles that met the inclusion criteria for this systematic review.

In the first search stage, a total of 247 papers were found. After the removal of duplicate papers, the titles and publication dates were rechecked to ensure that they met the initial eligibility criteria. Subsequently, the abstracts of 40 articles were screened to check if they were relevant to the research question. After careful review, a total of 17 articles were identified. Figure 1 shows how the studies were excluded at various stages using the PRISMA flow diagram.

### Quality Assessment of Eligible Trials

Table 3 shows the results of the NHLBI assessment. Six observational studies were rated as fair quality, while the rest were good quality studies.

**Table 3:** NHLBI quality assessment of the observational cohort studies.

**Study (years)**	**Question No**.												**TY**
	**1**	**2**	**3**	**4**	**5**	**6**	**7**	**8**	**9**	**10**	**11**	**12**	
De Souza Menezes et al. (22)	Y	Y	Y	Y	N	Y	Y	Y	Y	Y	Y	Y	**11**
Leite et al. (23)	Y	Y	N	Y	N	Y	Y	Y	Y	Y	U	Y	**9**
Ross et al. (24)	Y	Y	Y	Y	N	Y	Y	Y	Y	Y	U	Y	**10**
Bagri et al. (10)	Y	Y	N	Y	N	Y	Y	Y	Y	Y	U	N	**7**
Bechard et al. (25)	Y	Y	Y	N	N	Y	Y	Y	Y	Y	U	Y	**9**
Nangalu et al. (26)	Y	Y	U	Y	N	Y	Y	Y	Y	Y	U	N	**8**
Ward et al. (27)	Y	Y	N	Y	N	Y	Y	Y	Y	Y	U	Y	**9**
Costa et al. (28)	Y	Y	Y	Y	N	Y	Y	Y	Y	Y	U	Y	**10**
Grippa et al. (29)	Y	Y	N	Y	N	Y	Y	Y	Y	Y	U	Y	**9**
Anton-Martin et al. (30)	Y	Y	Y	N	N	Y	Y	Y	Y	Y	U	Y	**9**
Irving et al. (31)	Y	Y	U	Y	N	Y	Y	Y	N	Y	U	N	**7**
Chaitra et al. (32)	Y	Y	Y	Y	N	Y	Y	Y	Y	Y	Y	Y	**11**
Sharma et al. (33)	Y	Y	Y	Y	N	Y	Y	Y	Y	Y	U	N	**9**
Afonso et al. (34)	Y	Y	N	Y	Y	Y	U	Y	Y	Y	U	N	**8**
Ventura et al. (35)	Y	Y	Y	Y	N	Y	Y	Y	Y	Y	U	Y	**10**
Solana et al. (36)	Y	Y	U	Y	N	Y	Y	Y	Y	Y	U	N	**8**
Xiong et al. (37)	Y	Y	N	Y	N	Y	Y	Y	Y	Y	U	N	**7**

*N, No; NA; not applicable; U, unclear; Y, yes; TY, total yes*.

### Characteristics of Included Studies

A total of 17 studies involving 10,638 children were included in our systematic review (Table 4). Three studies were conducted in the developed countries, and 11 studies were conducted in the developing category; three studies were conducted across multiple countries and thus were categorized under a developed countries category because most of the countries included are developed. Mostly BMI methods were used to define the nutritional status, while the other anthropometric measurement was not utilized insufficiently.

**Table 4:** Summary of included studies.

**Study** **(years)**	**Country/** **Income Group**	**Design**	**Total N** **(UN: NN)**	**Age range**	**Patient cohort**	**BMI categories**	**Outcomes reported**			
							**Mortality**	**PICU LOS**	***MV usage***	***MV length***
De Souza Menezes et al. (22)	Brazil/Developing	Pros. cohort	369 (175: 192)	≤ 18y	Mixed	**Underweight:** < -2 z **Normal weight:** −2_≦+2 z	√	√	√	√
Leite et al. (23)	Brazil/Developing	Pros. cohort	221 (117: 104)	≤ 18y	Mixed	**Underweight:** < -2 z **Normal weight:** < −2 z	√	√		
Ross et al. (24)	US/ Developed	Ret. cohort	4,459 (819: 3,740)	≤ 18y	Sepsis	**Underweight:** <5% **Normal weight:** 5–85%	√	√	√	√
Bagri et al. (10)	Indian/ Developing	Ret. cohort	332 (190: 142)	1 m−15 y	Mixed	**Underweight:** < - 2 z **Normal weight:** −2_ <0 z	√	√	√	√
Bechard et al. (25)	Multiple countries	Pros. cohort	1,170 (291: 879)	1 m−18 y	Mixed	**Underweight:** < -2 z **Normal weight:** −2_≦1 z	√	√		
Nangalu et al. (26)	Indian/Developing	Pros. cohort	400 (154: 246)	1 m−14 y	Mixed	**Underweight:** <3 z **Normal nourished:** ≥3 z	√	√	√	√
Ward et al. (27)	Multiple countries	Pros. cohort	205 (40: 165)	≤ 18 y	Mixed PARDS	**Underweight:** <1.89 **Normal weight:** 1.89–1.04 z	√			√
Costa et al. (28)	Brazil/Developing	Ret. cross-sectional	881 (165: 716)	≤ 18 y	Mixed	**Underweight:** < -2 z **Normal weight:** >+2 z	√	√	√	
Grippa et al. (29)	Brazil/Developing	Pros. cohort	72 (15: 57)	1 m−15 y	Mixed	**Underweight:** ≦−2 z **Normal weight:** ≥-2 and ≤ 1 z			√	√
Anton-Martin et al. (30)	US/ Developed	Ret. cohort	447 (120: 327)	≤ 18 y	ECMO	**Underweight:** < -2 z **Normal weight:** −2_ ≦+2 z	√		√	√
Irving et al. (31)	Multiple countries	Pros. cohort	264 (126: 138)	≤ 18 y	Mixed sepsis	**Underweight:** < -1 z **Normal weight:** −1_≦ 1 z		√	√	
Chaitra. et al. (32)	India/Developing	Pros. cohort	41 (14: 27)	1 m−18 y	Mixed	**Underweight:** < -2 z **Normal weight:** ≥-2 and ≤ 1 z	√	√	√	
Sharma et al. (33)	US/Developed	Ret. cohort	1,107 (217: 890)	1 m−18 y	Mixed	**Underweight:** <5% **Normal weight:** 5–95%	√	√	√	√
Afonso et al. (34)	Brazil/Developing	Ret. cohort	36 (12: 24)	1–18 y	Sold tumor	**Underweight:** < -1 z **Normal weight:** ≥-1 and ≤ 1 z		√	√	
Ventura et al. (35)	Brazil/Developing	Pros. cohort	199 (33:133)	<15y	Mixed	**Underweight:** < -2 z **Normal weight:** −2 _ ≦+2 z	√	√		
Solana et al. (36)	Spanish/Developing	Pros. cross-sectional	97 (40: 57)	1 m−16 y	Mixed	**Underweight:** ≦−2 z **Normal weight:** >-2 _ < +2 z		√		
Xiong et al. (37)	China/Developing	Pros. cohort	240 (66:138)	≤ 14 y	Mixed	**Underweight:** ≦- 2 z **Normal weight:** 0_ < -2 z	√	√	√	√

*UN, undernutrition; NN, normal nutrition; N, number; BMI, body mass index; PIC, pediatric intensive care unit; LOS, length of stay; MV, mechanical ventilation; Pro, prospective; Ret, retrospective*.

Based on patient cohort diagnosis, 14 studies consisted of mixed PICU cohorts (including one sepsis study), and four studies consisted of specialized medical or surgical cohorts (pediatric acute respiratory distress syndrome (PARDS), extracorporeal membrane oxygenation (ECMO), sepsis, and solid tumor). Studies utilized our *a priori* cutoffs for BMI classification except for four studies (26, 27, 31, 34). Thus, we utilized each study's method of classification to define the weight categories; there were 8,044 (75.61%) normal weight and 2,594 (24.38%) underweight patients. Of the studies, 11 trials included participants up to 18 years of age (*n* = 1,330), while the remaining studies set the maximum age at 16 years or less (10, 26, 29, 35–37).

### Outcomes

#### Mortality

Eleven meta-analysis studies with 2,688 UN and 6,468 NN individuals were used to investigate the association between UN and death incidence in the PICU population. In this meta-analysis, the estimated mortality incidence was significant heterogeneity; therefore, the calculated RD cannot be estimated (RD = 0.02, *P* = 0.05, 95% CI = 0.00−0.03, chi-square *P* <0.0001, *I*^2^ = 78%). The sensitivity analysis was utilized to calculate a valid RD summary for mortality incidence in critically ill children. The RD was 5% significantly higher in UN subjects than on NN (RD = 0.05, *P* = 0.0005, 95% CI = 0.02–0.08, chi-square *P* = 0.18, *I*^2^ = 33%) after excluding De Souza Menezes et al. (22), Leite et al. (23), Ward et al. (27), and Xiong et al. (37).

**Figure 2:**
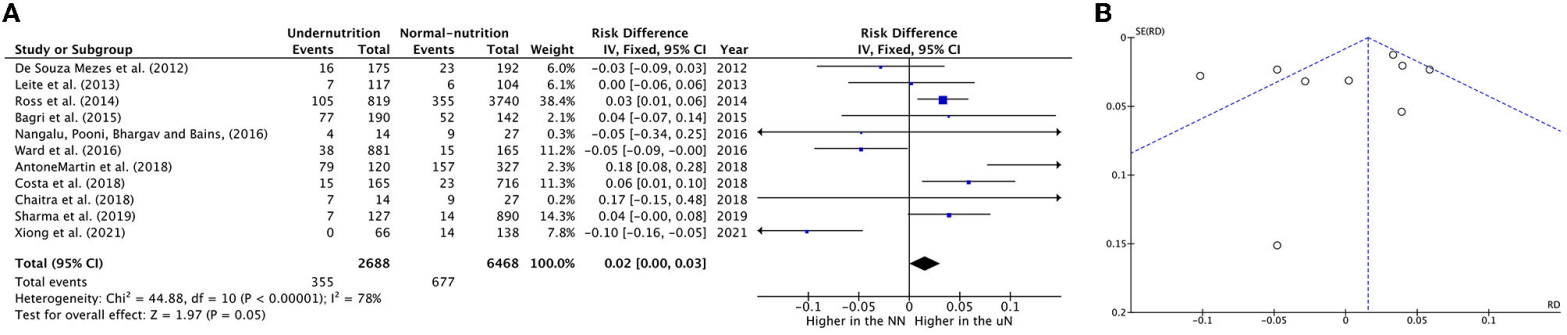
Forest plots of pooled **(A)** estimation RD of mortality incidence between PICU patients with undernutrition and normal nutrition. **(B)** Funnel plots assessing for publication bias in studies reporting estimation mortality RD between undernutrition and normal nutrition. CI, confidence interval.

The forest plot was described as having UN cases significantly decreased mortality incidence in two studies (27, 37), while four out of eleven studies described increased significance in mortality incidence on UN children, which was between 3 and 18 percentiles (24, 28, 30, 33); for more details, refer to Figure 2A.

Visual analysis suggested a non-publication bias in the results from the funnel in Figure 2B.

The clinical heterogeneity analysis exploring this outcome was regulated. The pooled UN definition analysis could not be applied to this outcome, as only one study fund had an atypical UN definition (z_score_ < -1) subgroup. The other heterogeneity analysis factors have remained significantly homogeneous in each group; thus, the RD calculation in these subgroups is unreliable, except for the developing country group (*I*^2^ = 23%, *P* = 0.26). While the subgroups' heterogeneity differences in the last three factors in Table 5 were shown to be non-significantly homogeneous. The RD pooled from the studies that define undernutrition by ≤ 1 zscore, and the studies with upper age range was ≤ 16 years revealed a non-significantly 4–5% reduction in mortality incidence in UN compared to NN. When mixed diagnoses group was represented, there was a equal in RD on UN than NN (RD 0.00, *P* > 0.05) (For more details, refer to Table 5).

**Table 5:** Results of heterogeneity test for the risk difference (RD) meta-analysis of UN vs. NN and mortality.

**Mortality incidence (Number of studies)**	**Subjects (*n*) UN/NN**	**Pooled RD [95% CI] Random Effect**	**Heterogeneity**	
			**Subgroup (P from Cochran Q)**	**Subgroup Difference**
**Definition of undernutrition:**				
< -2 z_score_ or 5th% (11)	1,807/6,303	0.02 [−0.02, 0.06]	*I*^2^ = 75%, *P =* <0.0001	*I*^2^ = 80.2%, *P* = 0.02
< -1 z_score_ (1)	881/165	−0.05 [−0.09, −0.00]	*I*^2^ = N/A, *P* = N/A	
**Upper range age accepted:**				
Up to 18 years (9)	2,418/6,161	0.03 [−0.01, 0.06]	*I*^2^ = 73%, *P* = 0.0006	*I*^2^ = 26.1%, *P* = 0.24
Up to 16 years (4)	270/307	−0.04 [−0.15, 0.07]	*I*^2^ = 63%, *P* = 0.07	
**Diagnostic type:**				
Mixed diagnose (9)	868/2,236	0.00 [−0.05, 0.05]	*I*^2^ = 72%, *P* = 0.0007	*I*^2^ = 0%, *P* = 0.45
Specific diagnose (4)	1,820/4,232	0.04 [−0.04, −0.13]	*I*^2^ = 89%, *P* = <0.0001	
**Economical country level:**				
Developed (6)	2,013/5,260	0.01 [−0.05, 0.07]	*I*^2^ = 89%, *P* = <0.0001	*I*^2^ = 0%, *P* = 0.81
Developing (7)	675/1,208	0.02 [−0.02, −0.6]	*I*^2^ = 23%, *P* = 0.26	

*UN, undernutrition; NN, normal nutrition; n, number; CI, confidence interval; P, P-value; Q, chi-square test*.

#### Use of MV

**Figure 3:**
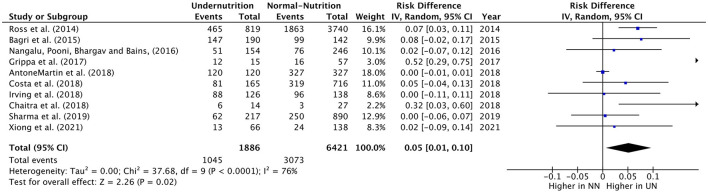
Forest plots of pooled estimation risk difference of MV usage between UN and NN patients. CI, confidence interval.

The RD of MV usage in PICU cases was estimated from teen eligible studies, with 1**,**886 UN and 6**,**421 NN individuals. The pooled RD of MV usage was significantly lower in UN by 5% (RD = 0.05, *P* = 0.02, 95% CI: 0.01–0.10, chi-square *P* <0.0001, *I*^2^ = 76%). As significant heterogeneity was observed, the sensitivity analysis was applied, so it was excluded Anton-Martin et al. (30) from the meta-analysis. As this study was conducted in ECMO patients, 100% of the sample was in MV (Figure 3). This lead to an increase in the estimated RD of MV used within UN critical ill children than NN within sensitive analysis (RD = 0.07, *P* = 0.02, 95% CI: 0.01–0.12, chi-square *P* = 0.003, *I*^2^ = 65%).

All the studies included showed a slight increase or non-difference in UN intubation cases in PICU, except for Grippa et al. (29), and Chaitra et al. (32), which showed a 52% and 32% UN used MV, significantly higher than NN cases.

The definition of UN difference subgroup analysis could not be applied to this outcome, as only one study was in the < -1 z_score_ group. The z-score < -2 or in the 5%, mixed diagnostic type and developing subgroups studies showed a considerable significant 6%–12% increase in UN, comparable to NN in the RD of usage MV. Regarding the heterogeneity analysis that was conducted in need of MV RD, the homoogeneity in subgroup diffrence was substantially non-significant, except for the economical country level, which was a significant heterogeneity sample (chi-square *P* = 0.09, *I*^2^ = 64%; refer to Table 6 for more information).

**Table 6:** Results of sensitivity analyses for the risk difference (RD) meta-analysis of UN vs. NN and MV usage.

**MV Usage (Number of studies)**	**Subjects (*n*) UN/NN**	**Pooled RD [95% CI] Random Effect**	**Heterogeneity**	
			**Subgroup (P from Cochran Q)**	**Subgroup Difference**
**Definition of undernutrition**				
< -2 z_score_ or 5th% (10)	1,732/6,175	0.06 [0.01, 0.10]	*I*^2^ = 79%, *P* = <0.0001	*I*^2^ = 0%, *P* = 0.53
< -1 z_score_ (1)	154/246	0.02 [−0.07, 0.12]	*I*^2^ = N/A, *P* = N/A	
**Upper range age accepted**				
Up to 18 y (8)	1,615/6,084	0.03 [−0.01, 0.07]	*I*^2^ = 66%, *P* = 0.008	*I*^2^ = 44.9%, *P* = 0.18
Up to 16 y (4)	271/337	0.18 [−0.03, 0.38]	*I*^2^ = 86%, *P* = 0.008	
**Diagnostic type**				
Mixed diagnose (9)	947/2,354	0.07 [0.00, 0.14]	*I*^2^ = 68%, *P* = 0.002	*I*^2^ = 0%, *P* = 0.42
Specific diagnose (3)	939/4,067	0.03 [−0.04, 0.10]	*I*^2^ = 92%, *P* = 0.0005	
**Economical country level**				
Developed (5)	1,282/5,095	0.02 [−0.02, 0.07]	*I*^2^ = 75%, *P* = 0.007	*I*^2^ = 64%, *P* = 0.09
Developing (7)	604/1,326	0.12 [0.02, 0.21]	*I*^2^ = 74%, *P* = 0.002	

*UN, undernutrition; NN, normal nutrition; n, number; MV, mechanical ventilation; CI, confidence interval; P, P-value; Q, chi-square test*.

#### LOS in PICU

**Figure 4:**
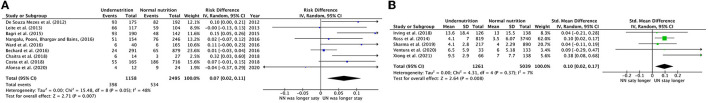
Forest plots of pooled **(A)** estimation risk difference, **(B)** standardized mean difference of PICU LOS between undernutrition and normal nutrition patients. CI, confidence interval.

The RD of LOS in the PICU was calculated from nine eligible studies, which included 1,158 UN cases and 2,495 controls. The RD of staying longer in PICU in UN was significantly 7%, which was comparable to NN children. Figure 4A provides the summary statistics for the RD of staying longer in the PICU, regardless of the way to classify the number of longer stay days between the studies. Therefore, the summary showed significant moderate heterogeneity (chi-square *P* = 0.05, *I*^2^ = 48%). Afonso et al. (34) was the only study that showed a non-significant reduction in UN in critically ill children by 4% compared to NN.

The five studies that reported PICU LOS as a mean duration, in days, were pooled in a meta-analysis (Figure 4B). There was a minor significant impact in pooled mean PICU LOS when comparing UN to NN patients (SMD = 0.10, *P* = 0.008, 95% CI = 0.02–0.17 days, chi-square *P* = 0.37, *I*^2^ = 7%). The heterogeneity analysis could only devote to the upper range age factor, as both subgroups conducted more than two studies. The studies that define the UN with < -2 zscore or 5th percentile, except for the upper range of ≤ 18 age, with mixed diagnoses, conducted in developing countries and stay in PICU for > 6 days showed a slightly significant increased RD in UN compared to NN children (RD = 0.06, 0.06, 0.06, 0.05, and 0.05, respectively).

In sensitivity analysis, it was found that UN patients stay longer than NN patients 10%–17% in the subgroup studies that were deifine the undernutriton case with < -1 zscore, included only specific diagnosis, or define the long stay in PICU ≧ 3 days (Table 7). Publication bias was not assessed due to a limited number of studies.

**Table 7:** Results of sensitivity analyses for the risk difference (RD) meta-analysis of UN vs. NN and PICU LOS.

**PICU LOS (Number of studies)**	**Subjects (*n*) UN/NN**	**Pooled RD [95% CI] Random Effect**	**Heterogeneity**	
			**Subgroup (P from Cochran Q)**	**Subgroup Difference**
**Definition of undernutrition**				
< -2 z_score_ or 5th% (9)	1,106/2,306	0.06 [0.01, 0.12]	*I*^2^ = 56%, *P* = 0.03	*I*^2^ = 0%, *P* = 0.58
< -1 z_score_ (3)	52/189	0.10 [−0.01, 0.20]	*I*^2^ = 0%, *P* = 0.38	
**Upper range age accepted**				
Up to 18 y (8)	814/2,107	0.06 [0.01, 0.11]	*I*^2^ = 45%, *P* = 0.09	*I*^2^ = 0%, *P* = 0.02
Up to 16 y (4)	344/388	0.08 [−0.04, 0.21]	*I*^2^ = 69%, *P* = 0.07	
**Diagnostic type**				
Mixed diagnose (9)	1,106/2,306	0.06 [0.01, 0.12]	*I*^2^ = 56%, *P* = 0.03	*I*^2^ = 0%, *P* = 0.58
Specific diagnose (3)	52/189	0.10 [−0.01, 0.20]	*I*^2^ = 0%, *P* = 0.38	
**Economical country level**				
Developed (3)	331/1,044	0.05 [−0.05, 0.15]	*I*^2^ = 66%, *P* = 0.09	*I*^2^ = 0%, *P* = 0.59
Developing (9)	827/1,451	0.08 [0.02, 0.13]	*I*^2^ = 27%, *P* = 0.23	
**Definition long duration**				
>6 days (8)	969/2,276	0.05 [0.00, 0.10]	*I*^2^ = 41%, *P* = 0.12	*I*^2^ = 26.8%, *P* = 0.24
>3 days (4)	189/219	0.17 [−0.02, 0.36]	*I*^2^ = 47%, *P* = 0.17	

*UN, undernutrition; NN, normal nutrition; n, number; LOS, length of stay; PICU, pediatric intensive care unit; CI, confidence interval; P, P-value; Q, chi-square test*.

#### Prolonged Use of MV

**Figure 5:**
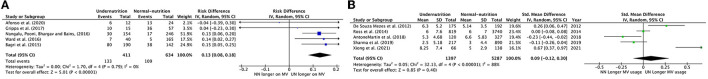
Forest plots of pooled **(A)** estimation risk difference, **(B)** standardized mean difference of prolonged use of MV between undernutrition and normal nutrition PICU patients. CI, confidence interval.

Five meta-analysis studies with 411 UN and 634 NN individuals were used to investigate the association between nutrition status and MV duration. Figure 5A shows that 13% of UN cases were significantly more likely to stay longer than 7 days on MV than NN (RD = 0.13, *P* < 0.0001, 95% CI = 0.08–0.18; chi-square *P* = 79, *I*^2^ = 0.0%). Pooled RD in Bagri et al. (10), Nangalu et al. (26), and Ward et al. (27) represent a significantly higher risk of MV usage longer for UN critically ill children compared to NN (15, 13, 14%; respectively).

Five studies were included in the meta-analysis that reported prolonged MV usage as an SMD duration per day (Figure 5B). There was a non-significant small effect of UN states on prolonged MV usage compared to NN states (SMD = 0.09, *P* = 0.40, 95% CI −0.12 to 0.30 days, chi-square *P* < 0.0001, *I*^2^ = 88%), where only Xiong et al. (37) showed a significant considerable prolonging intubation period for UN vs. NN, 67%. However, to reduce the heterogenity between the studies, de Souza Menezes et al. (22), Anton-Martin et al. (30), and Xiong et al. (37) were excluded from the Forest plots of pooled analysis, which result in regress the heterogeneity to I^2^ 40% (chi-square *P* < 0.0001), and the SMD was −0.04 insignificantly.

In the SMD of prolonged MV usage outcome, only the diagnostic type of heterogeneity analysis was applicable to conduct. It demonstrated that UN patients in the mixed diagnosed subgroup had a small effect on staying in MV for longer than NN patients (SMD = 0.26, *P* = 0.23, 95% CI = 0.16–0.68 days, chi-square *P* < 0.0001, *I*^2^ = 92%). In contrast, within specific diagnoses, there was a little effect between UN and NN patients (SMD = 0.10, *P* = 0.41, 95% CI = −0.32–0.13 days, chi-square *P* = 0.04, I^2^ = 76%).

Due to the limited number of research, publication bias was not examined.

## Discussion

In our systematic review, we found a slightly significant risk for UN children to stay longer in critical care units by 7% and a mild significant risk of prolonged MV usage (13%) compared to NN children. Prolonged MV usage trend was also observed similarly in all subgroups analyzed. When we focused on pooled studies of both developing countries and younger children's samples, we found that the highest risk needs MV odds in UN patients relative to NN.

However, in contrast to earlier findings, significant RD evidence of mortality incidence was detected between both nutritional status groups. However, we cannot rely on this result as the heterogeneity was high. Therefore, we excluded studies with a non-significant result to test the sensitivity analysis, which were studies by Leite et al. (23), Ward et al. (27), Anton-Martin et al. (30), Costa et al. (28), and Xiong et al. (37). Surprisingly, we found that mortality incidence in UN PICU was 5% higher than in the NN on a homogeneous sample. The five excluded studies had only limited power because most of the studies were small.

The association between UN and inferior PICU outcomes might be explained by a depleted metabolic store with a high catabolic state, low nutritional intake or decreased absorption efficacy (38, 39), loss of muscle mass and strength affecting respiratory function (40, 41), as well as impaired immunity and high oxidative stress associated with delayed wound healing and increased infection risks (40, 42). Thus, in future studies nutrition states must be evaluated during the whole PICU period rather than at admission.

Children in the critical care unit are at risk of having their nutritional status deteriorating due to their disease or barriers to nutrient delivery (43), further worsening their prognosis (42). Appropriate nutritional interventions for UN patients can decrease their incidence of mortality, which is similarly found in the study by Sharma et al. (33); the intervention group had a decreased mortality incidence as a result of appropriate nutritional interventions.

The lack of differences in clinical outcomes demonstrated in our review differs from a systematic review conducted by Costa et al. (13) and a meta-analysis review conducted by Toh et al. (12). These prior reviews either did not demonstrate the association or non-significant association between mortality, PICU LOS, and admission nutrition status. This is likely due to the high heterogeneity within studies and differences in the classification of UN in their included studies. However, the significant results in our review may possibly be due to verified heterogeneity analysis and a more restricted methodology. Only three of the included studies utilized upper z-score cutoffs than our *a priori* z-score cutoffs, which were based on different growth reference studies (27, 31, 34). The remaining studies used either BMI percentiles or *a priori* z-score cutoffs that we adopted from WHO (17).

We were only able to analyze PICU LOS and extended MV in UN individuals in ≦ −2 z_score_ and ≦ −1 z_score_ pooled due to the minimal variability in nutrition status definitions across studies. UN children are at risk of staying longer in PICU compared to NN children when UN defined from < -1 z_score_ by 4% than UN standard classification. This may indicate a classification overlap. In most of the studies, a patient who was between the −1 and −2 SD line would have been classified as NN, but Ward et al. (27), Irving et al. (31), and Afonso et al. (34) counted them as UN patients. Given that our sensitivity analysis found discrepancies in results, it appears that we, as a research community, need to standardize the classification of critically ill children's nutritional status.

However, there was significant heterogeneity in defining upper accepting age range subgroup and mortality incidence, MV need, and PIC LOS. But, when the ≦16 years patient cohorts were analyzed alone, we revealed lower mortality odds in UN patients relative to NN. In contrast, the risk of using MV outcome was mildest higher in UN compared to NN children. The explanation of that significant moderate heterogeneity was still even after subgroup the define upper accepting age range is a reflection of the growth cycle. As in infants, toddlers, and the last 2 years of adolescent ages, the body composition especially muscle mass can be fluctuating (44); besides, evidence suggests that when low muscle mass is present, the risk of death in critically ill patients is increased (45). This led us to the hypothesis that subgroups must be categorized according to narrowing age, i.e., infancy, toddler, childhood, and adolescence.

We further explored this influence by heterogeneity analysis including mixed patient cohorts, which revealed a 7% higher risk of MV usage in UN compared to NN patients. It was logical to exclude the Anton-Martin et al. (30) study to get a sensitivity analysis as it was focused only on ventilated patient population (ECMO cases). However, exclusion was impossible in this situation as the specific diagnosis group contained only Anton-Martin et al. (30) and Ross et al. (24). As a result, we can rule out the possibility of heterogeneous research populations having an impact on our major findings. In certain research, chronic diseases are known to cause UN (46); therefore, it is important to know the patient's diagnostic history and frequency of hospitalization.

In addition, it was analyzed to explore the prospective influence of economical country status on the effect of the UN on PICU outcomes. Analyzing mortality incidence in both developing and developed countries showed no significant RD, which was relatively higher in UN compared to NN patients. In contrast, the estimated RD of MV usage incidence in UN patients were ten time higher in developing countries than in developed countries. Thus, we can hypothesize that the lack of impact distinction is due to a lack of literature undertaken in underdeveloped countries (47). Furthermore, poorer results in lower-income nations may be due to differences in PICU resources between countries (48, 49), which may not have been apparent in the papers we reviewed.

The last heterogeneity analysis in our review was the number of days to define long period even in PICU staying. When we compared the pooled results from studies that used ≦3 days as a cutoff to describe their sample longer stay in PICU to ≦7 days pooled result, we found that UN had the highest risk of prolonged stay in PICU than NN in ≦7 days group. Despite this, this meta-analysis of the stay prolonged in MV included RDs had only limited power because most of the subgroups have one study.

### Potential Limitations to the Review Process

The methodological quality of the included studies varied. Their limitations affected the analysis of the LOS in the PICU and the prolonged MV usage. In the included studies, these varied between 3 and 7 days. In addition, the meta-analysis could not differentiate between the types of MV used, which can result in substantial variations in the RD (50). There were also limitations among the populations studied, as some of them had a wider age range that included early adults. All these variations could have resulted in overlaps in the results between the studies. Furthermore, due to the small number of papers included, our systematic review may be limited by publication bias in most of the outcomes.

Finally, the difficulty of using BMI to distinguish between fat and muscle mass, as well as the effect of fluid imbalances and linear development anomalies, restricts its use as a malnutrition indicator (31, 51, 52). Arm anthropometry, such as mid-upper arm circumference or calf circumference, has been demonstrated to be a rapid, effective measure that may be better at representing body composition than BMI (53, 54), and is an alternative nutritional evaluation to investigate for future research. Despite this, BMI is widely employed because of its relative ease of measurement and computation, allowing rapid nutritional assessment in large patient groups. Standardization of BMI classifications in future research, perhaps combined with additional nutritional status indicators, may aid in a better understanding of the relationship between malnutrition and outcomes in critically ill children.

## Conclusion

This systematic review established a significant association between UN status at PICU admission and clinical outcomes such as mortality incidence, need for MV, PICU LOS, and prolonged stay in MV. All studies examined were observational in nature and were consistent in their use of the nutritional assessment parameters (anthropometry, i.e., BMI and W/H) in the pediatric intensive care unit. However, there was considerable variance in the classification of the UN between studies. Sensitivity analysis revealed that an increased risk of PICU stay, MV usage, and duration on MV may be associated with UN at the time of PICU admission, as assessed by BMI z-score. Additionally, the data suggest that the primary diagnosis for PICU admission and economical country level may influence clinical outcomes. This may imply the necessity for standardization of UN status classification based on hospital or PICU admission status, stratified by patients' diagnosis subgroups at admission, to facilitate future research elucidating the relationship between nutritional status and clinical outcomes in PICU patients.

## Data Availability Statement

The datasets presented in this study can be found in online repositories. The names of the repository/repositories and accession number(s) can be found in the article/supplementary material.

## Author Contributions

MA designed the study, interpreted the data, and wrote the manuscript. KB takes responsibility for the integrity of the data and the accuracy of the data analysis. Both authors reviewed and contributed to the manuscript drafted by MA. Both authors have read the manuscript, took part in the discussion, and agreed the final version.

## Conflict of Interest

The authors declare that the research was conducted in the absence of any commercial or financial relationships that could be construed as a potential conflict of interest.

## Publisher's Note

All claims expressed in this article are solely those of the authors and do not necessarily represent those of their affiliated organizations, or those of the publisher, the editors and the reviewers. Any product that may be evaluated in this article, or claim that may be made by its manufacturer, is not guaranteed or endorsed by the publisher.
